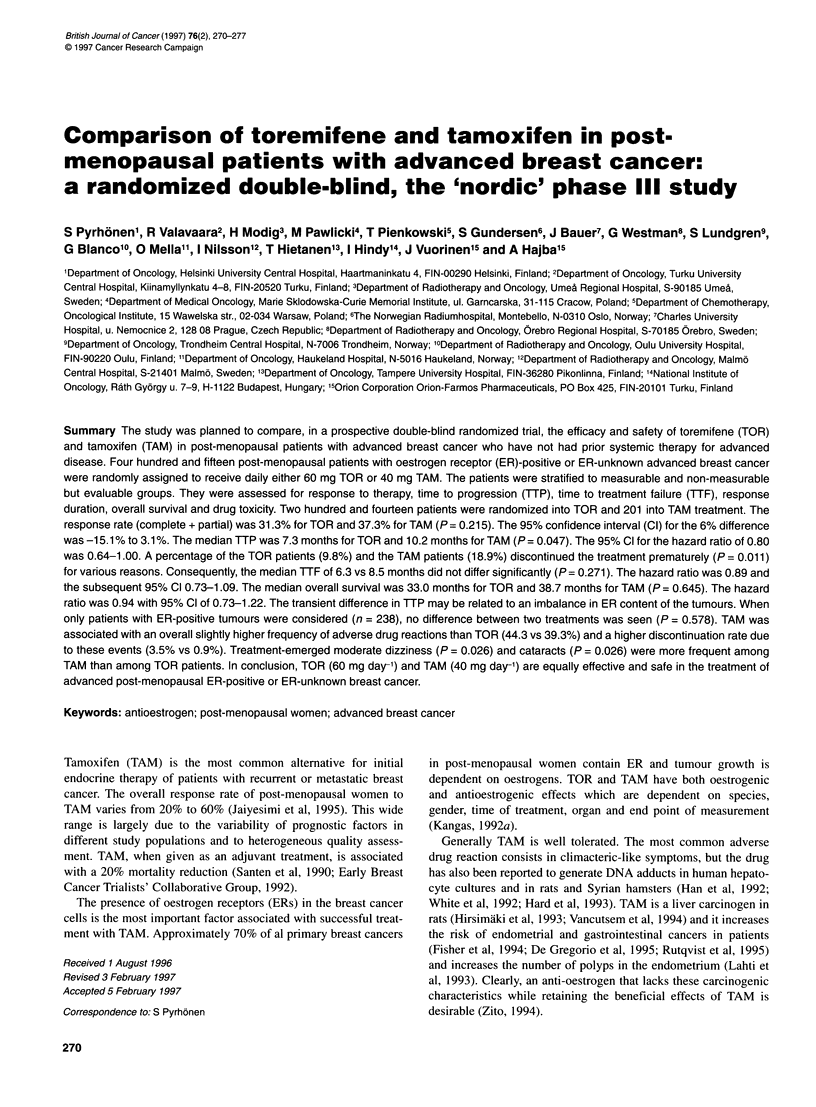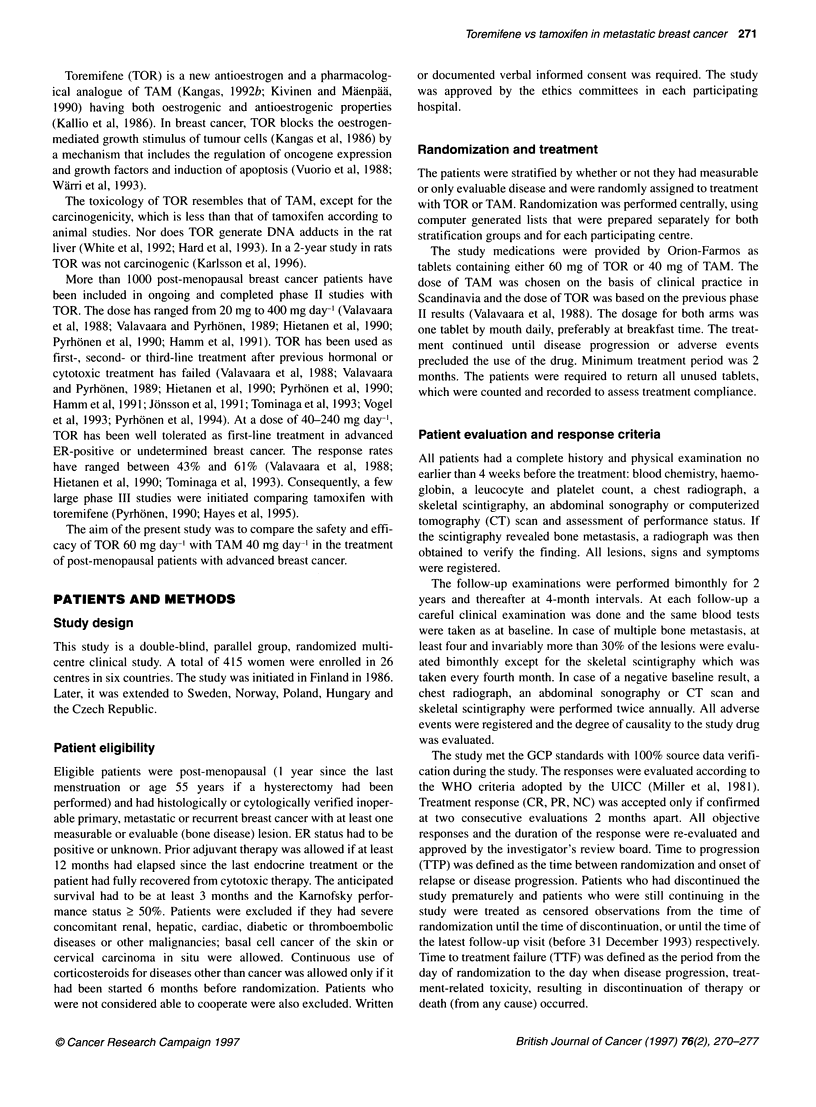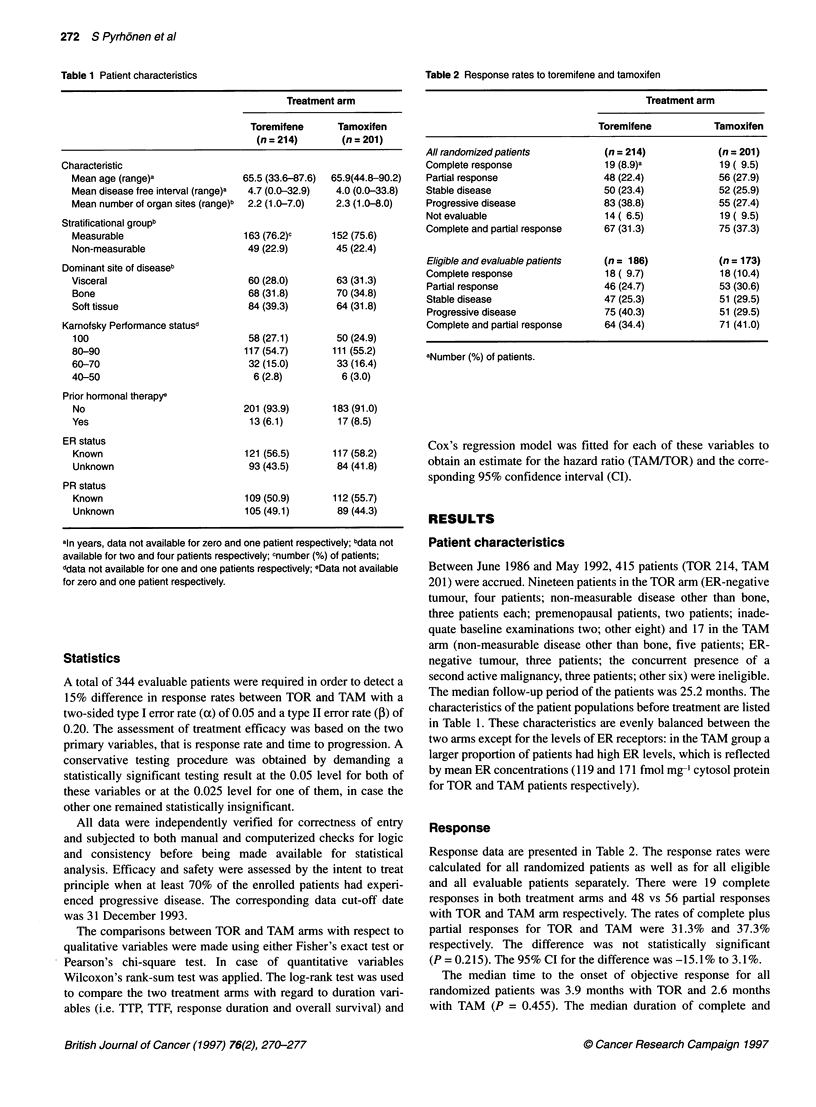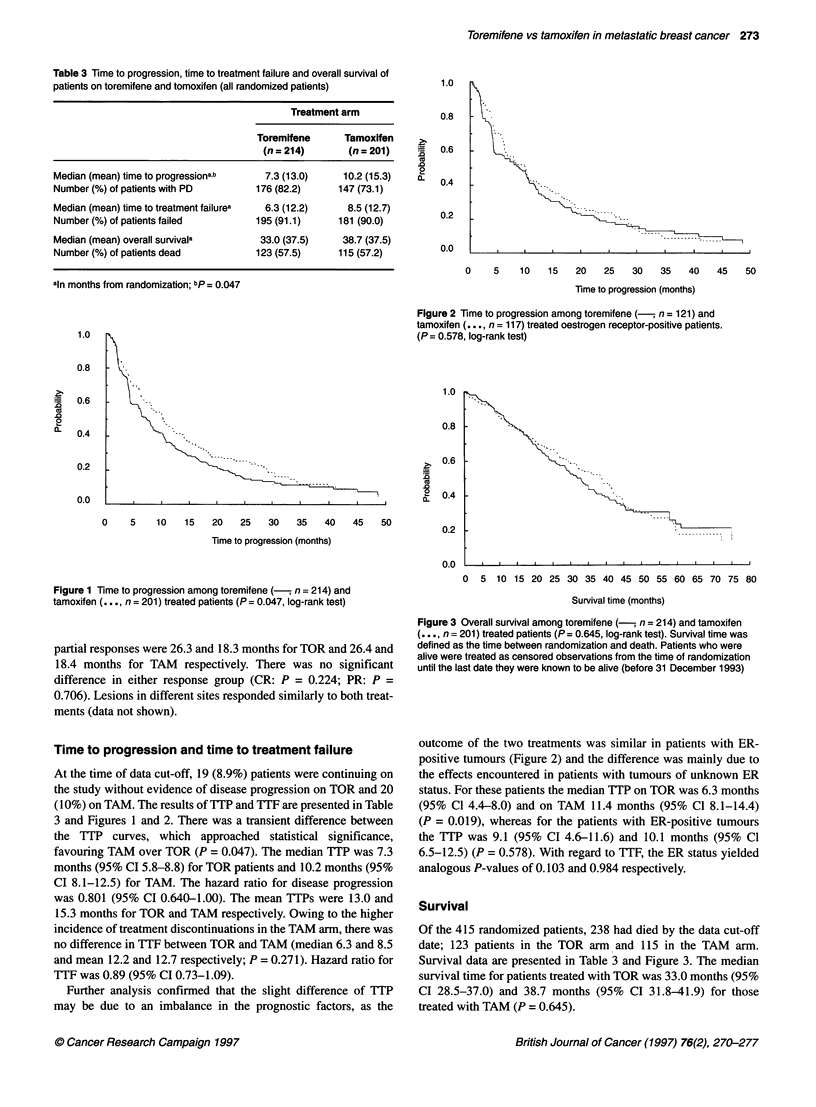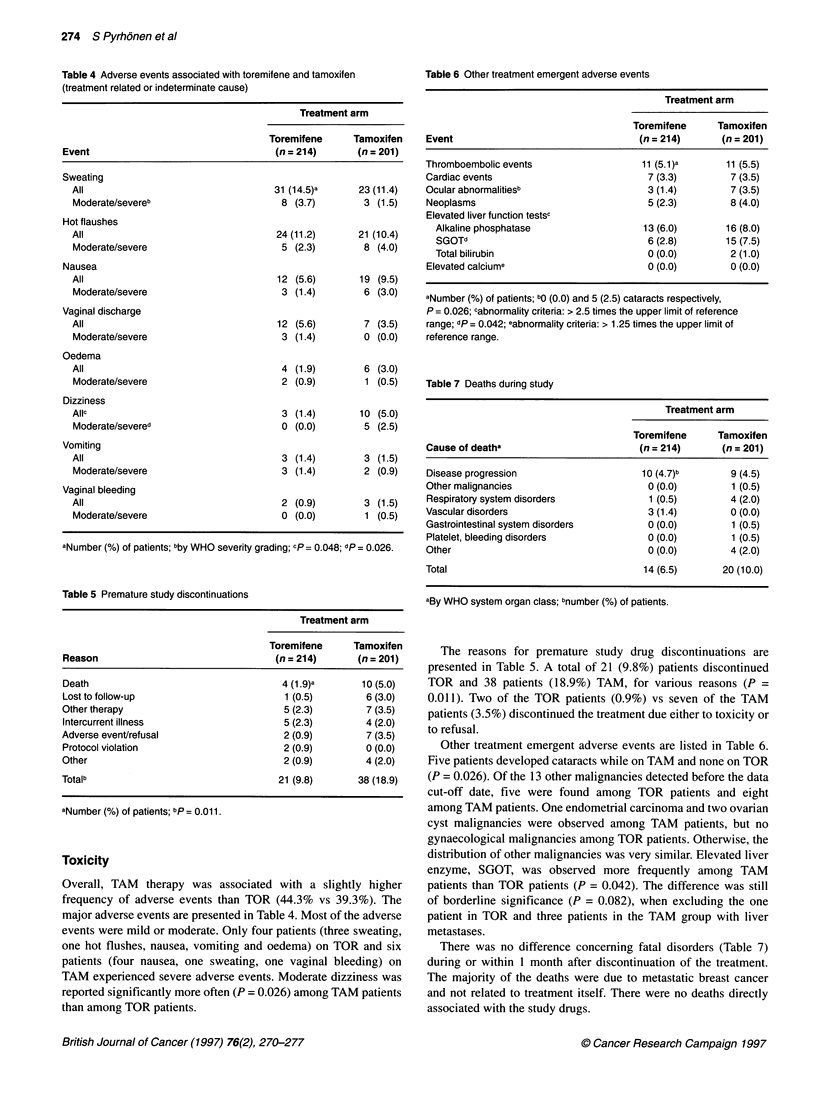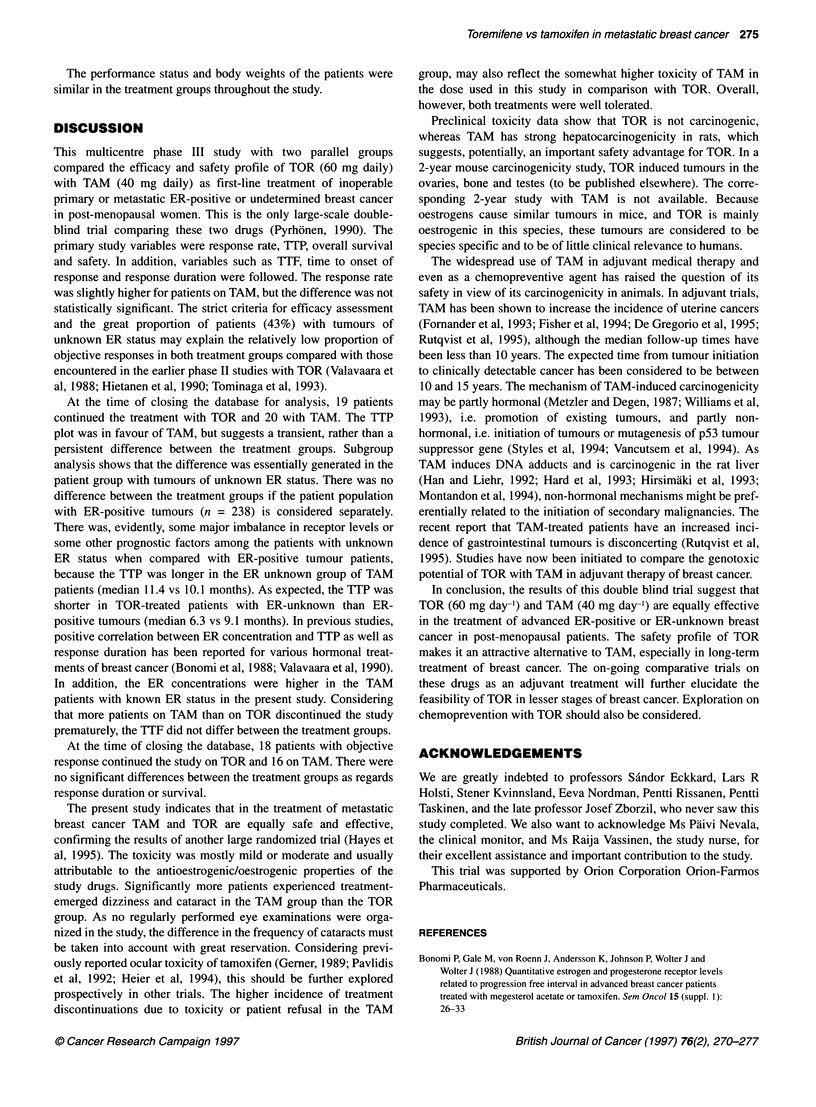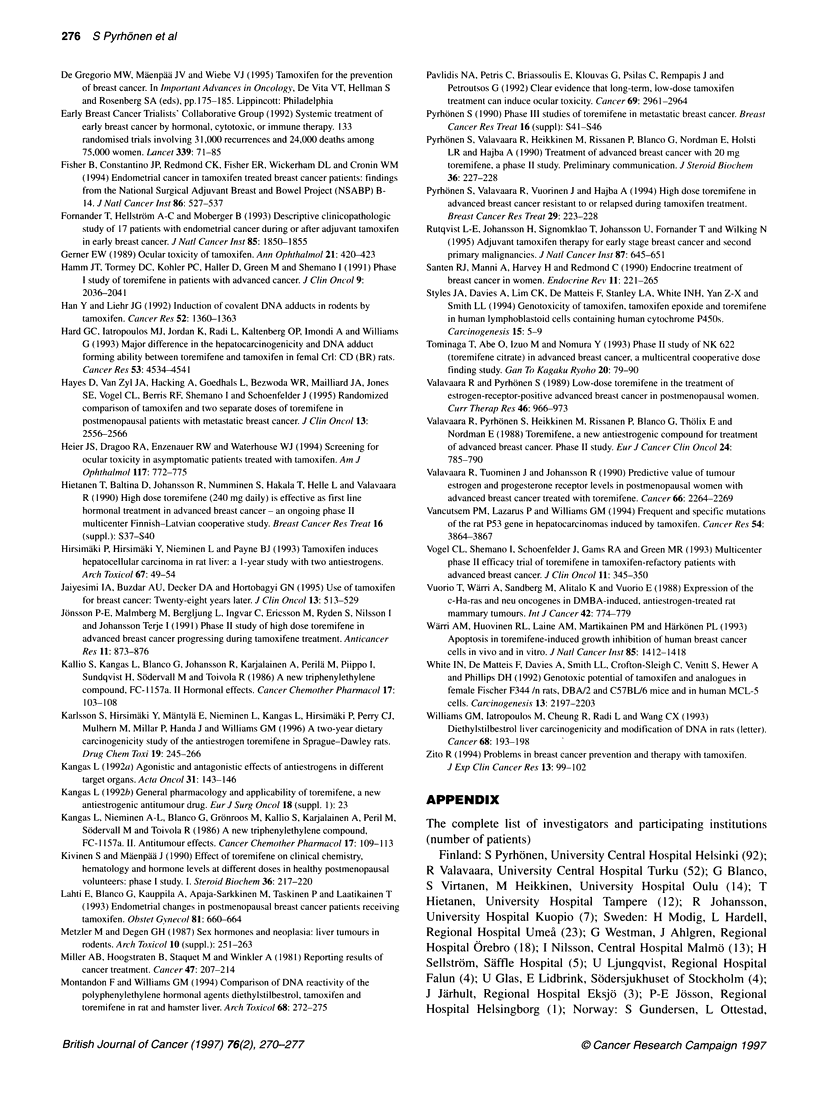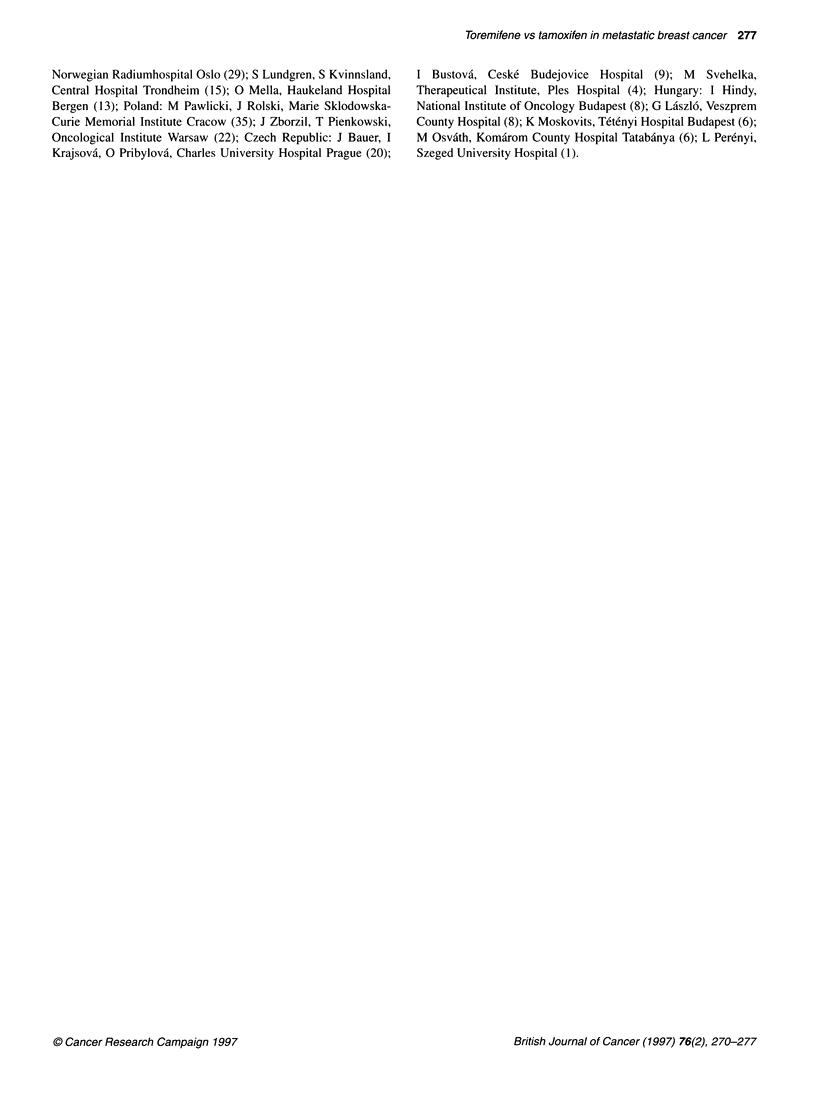# Comparison of toremifene and tamoxifen in post-menopausal patients with advanced breast cancer: a randomized double-blind, the 'nordic' phase III study.

**DOI:** 10.1038/bjc.1997.375

**Published:** 1997

**Authors:** S. PyrhÃ¶nen, R. Valavaara, H. Modig, M. Pawlicki, T. Pienkowski, S. Gundersen, J. Bauer, G. Westman, S. Lundgren, G. Blanco, O. Mella, I. Nilsson, T. Hietanen, I. Hindy, J. Vuorinen, A. Hajba

**Affiliations:** Department of Oncology, Helsinki University Central Hospital, Finland.

## Abstract

The study was planned to compare, in a prospective double-blind randomized trial, the efficacy and safety of toremifene (TOR) and tamoxifen (TAM) in post-menopausal patients with advanced breast cancer who have not had prior systemic therapy for advanced disease. Four hundred and fifteen post-menopausal patients with oestrogen receptor (ER)-positive or ER-unknown advanced breast cancer were randomly assigned to receive daily either 60 mg TOR or 40 mg TAM. The patients were stratified to measurable and non-measurable but evaluable groups. They were assessed for response to therapy, time to progression (TTP), time to treatment failure (TTF), response duration, overall survival and drug toxicity. Two hundred and fourteen patients were randomized into TOR and 201 into TAM treatment. The response rate (complete + partial) was 31.3% for TOR and 37.3% for TAM (P = 0.215). The 95% confidence interval (CI) for the 6% difference was -15.1% to 3.1%. The median TTP was 7.3 months for TOR and 10.2 months for TAM (P = 0.047). The 95% CI for the hazard ratio of 0.80 was 0.64-1.00. A percentage of the TOR patients (9.8%) and the TAM patients (18.9%) discontinued the treatment prematurely (P = 0.011) for various reasons. Consequently, the median TTF of 6.3 vs 8.5 months did not differ significantly (P = 0.271). The hazard ratio was 0.89 and the subsequent 95% CI 0.73-1.09. The median overall survival was 33.0 months for TOR and 38.7 months for TAM (P = 0.645). The hazard ratio was 0.94 with 95% CI of 0.73-1.22. The transient difference in TTP may be related to an imbalance in ER content of the tumours. When only patients with ER-positive tumours were considered (n = 238), no difference between two treatments was seen (P = 0.578). TAM was associated with an overall slightly higher frequency of adverse drug reactions than TOR (44.3 vs 39.3%) and a higher discontinuation rate due to these events (3.5% vs 0.9%). Treatment-emerged moderate dizziness (P = 0.026) and cataracts (P = 0.026) were more frequent among TAM than among TOR patients. In conclusion, TOR (60 mg day(-1)) and TAM (40 mg day(-1)) are equally effective and safe in the treatment of advanced post-menopausal ER-positive or ER-unknown breast cancer.


British Journal of Cancer (1997) 76(2), 270–277
© 1997 Cancer Research Campaign

### Comparison of toremifene and tamoxifen in post-menopausal patients with advanced breast cancer: a randomized double-blind, the 'nordic' phase III study

S Pyrhönen[1], R Valavaara[2], H Modig[3], M Pawlicki[4], T Pienkowski[5], S Gundersen[6], J Bauer[7], G Westman[8], S Lundgren[9], G Blanco[10], O Mella[11], I Nilsson[12], T Hietanen[13], I Hindy[14], J Vuorinen[15] and A Hajba[15]

[1]Department of Oncology, Helsinki University Central Hospital, Haartmaninkatu 4, FIN-00290 Helsinki, Finland; [2]Department of Oncology, Turku University Central Hospital, Kiinamyllynkatu 4–8, FIN-20520 Turku, Finland; [3]Department of Radiotherapy and Oncology, Umeå Regional Hospital, S-90185 Umeå, Sweden; [4]Department of Medical Oncology, Marie Sklodowska-Curie Memorial Institute, ul. Garncarska, 31-115 Cracow, Poland; [5]Department of Chemotherapy, Oncological Institute, 15 Wawelska str., 02-034 Warsaw, Poland; [6]The Norwegian Radiumhospital, Montebello, N-0310 Oslo, Norway; [7]Charles University Hospital, u. Nemocnice 2, 128 08 Prague, Czech Republic; [8]Department of Radiotherapy and Oncology, Örebro Regional Hospital, S-70185 Örebro, Sweden; [9]Department of Oncology, Trondheim Central Hospital, N-7006 Trondheim, Norway; [10]Department of Radiotherapy and Oncology, Oulu University Hospital, FIN-90220 Oulu, Finland; [11]Department of Oncology, Haukeland Hospital, N-5016 Haukeland, Norway; [12]Department of Radiotherapy and Oncology, Malmö Central Hospital, S-21401 Malmö, Sweden; [13]Department of Oncology, Tampere University Hospital, FIN-36280 Pikonlinna, Finland; [14]National Institute of Oncology, Ráth György u. 7–9, H-1122 Budapest, Hungary; [15]Orion Corporation Orion-Farmos Pharmaceuticals, PO Box 425, FIN-20101 Turku, Finland

**Summary** The study was planned to compare, in a prospective double-blind randomized trial, the efficacy and safety of toremifene (TOR) and tamoxifen (TAM) in post-menopausal patients with advanced breast cancer who have not had prior systemic therapy for advanced disease. Four hundred and fifteen post-menopausal patients with oestrogen receptor (ER)-positive or ER-unknown advanced breast cancer were randomly assigned to receive daily either 60 mg TOR or 40 mg TAM. The patients were stratified to measurable and non-measurable but evaluable groups. They were assessed for response to therapy, time to progression (TTP), time to treatment failure (TTF), response duration, overall survival and drug toxicity. Two hundred and fourteen patients were randomized into TOR and 201 into TAM treatment. The response rate (complete + partial) was 31.3% for TOR and 37.3% for TAM ($P = 0.215$). The 95% confidence interval (CI) for the 6% difference was –15.1% to 3.1%. The median TTP was 7.3 months for TOR and 10.2 months for TAM ($P = 0.047$). The 95% CI for the hazard ratio of 0.80 was 0.64–1.00. A percentage of the TOR patients (9.8%) and the TAM patients (18.9%) discontinued the treatment prematurely ($P = 0.011$) for various reasons. Consequently, the median TTF of 6.3 vs 8.5 months did not differ significantly ($P = 0.271$). The hazard ratio was 0.89 and the subsequent 95% CI 0.73–1.09. The median overall survival was 33.0 months for TOR and 38.7 months for TAM ($P = 0.645$). The hazard ratio was 0.94 with 95% CI of 0.73–1.22. The transient difference in TTP may be related to an imbalance in ER content of the tumours. When only patients with ER-positive tumours were considered ($n = 238$), no difference between two treatments was seen ($P = 0.578$). TAM was associated with an overall slightly higher frequency of adverse drug reactions than TOR (44.3 vs 39.3%) and a higher discontinuation rate due to these events (3.5% vs 0.9%). Treatment-emerged moderate dizziness ($P = 0.026$) and cataracts ($P = 0.026$) were more frequent among TAM than among TOR patients. In conclusion, TOR (60 mg day$^{-1}$) and TAM (40 mg day$^{-1}$) are equally effective and safe in the treatment of advanced post-menopausal ER-positive or ER-unknown breast cancer.

**Keywords:** antioestrogen; post-menopausal women; advanced breast cancer

Tamoxifen (TAM) is the most common alternative for initial endocrine therapy of patients with recurrent or metastatic breast cancer. The overall response rate of post-menopausal women to TAM varies from 20% to 60% (Jaiyesimi et al, 1995). This wide range is largely due to the variability of prognostic factors in different study populations and to heterogeneous quality assessment. TAM, when given as an adjuvant treatment, is associated with a 20% mortality reduction (Santen et al, 1990; Early Breast Cancer Trialists' Collaborative Group, 1992).

The presence of oestrogen receptors (ERs) in the breast cancer cells is the most important factor associated with successful treatment with TAM. Approximately 70% of al primary breast cancers in post-menopausal women contain ER and tumour growth is dependent on oestrogens. TOR and TAM have both oestrogenic and antioestrogenic effects which are dependent on species, gender, time of treatment, organ and end point of measurement (Kangas, 1992*a*).

Generally TAM is well tolerated. The most common adverse drug reaction consists in climacteric-like symptoms, but the drug has also been reported to generate DNA adducts in human hepatocyte cultures and in rats and Syrian hamsters (Han et al, 1992; White et al, 1992; Hard et al, 1993). TAM is a liver carcinogen in rats (Hirsimäki et al, 1993; Vancutsem et al, 1994) and it increases the risk of endometrial and gastrointestinal cancers in patients (Fisher et al, 1994; De Gregorio et al, 1995; Rutqvist et al, 1995) and increases the number of polyps in the endometrium (Lahti et al, 1993). Clearly, an anti-oestrogen that lacks these carcinogenic characteristics while retaining the beneficial effects of TAM is desirable (Zito, 1994).

*Received 1 August 1996*
*Revised 3 February 1997*
*Accepted 5 February 1997*

*Correspondence to:* S Pyrhönen

Toremifene (TOR) is a new antioestrogen and a pharmacological analogue of TAM (Kangas, 1992*b*; Kivinen and Mäenpää, 1990) having both oestrogenic and antioestrogenic properties (Kallio et al, 1986). In breast cancer, TOR blocks the oestrogen-mediated growth stimulus of tumour cells (Kangas et al, 1986) by a mechanism that includes the regulation of oncogene expression and growth factors and induction of apoptosis (Vuorio et al, 1988; Wärri et al, 1993).

The toxicology of TOR resembles that of TAM, except for the carcinogenicity, which is less than that of tamoxifen according to animal studies. Nor does TOR generate DNA adducts in the rat liver (White et al, 1992; Hard et al, 1993). In a 2-year study in rats TOR was not carcinogenic (Karlsson et al, 1996).

More than 1000 post-menopausal breast cancer patients have been included in ongoing and completed phase II studies with TOR. The dose has ranged from 20 mg to 400 mg day$^{-1}$ (Valavaara et al, 1988; Valavaara and Pyrhönen, 1989; Hietanen et al, 1990; Pyrhönen et al, 1990; Hamm et al, 1991). TOR has been used as first-, second- or third-line treatment after previous hormonal or cytotoxic treatment has failed (Valavaara et al, 1988; Valavaara and Pyrhönen, 1989; Hietanen et al, 1990; Pyrhönen et al, 1990; Hamm et al, 1991; Jönsson et al, 1991; Tominaga et al, 1993; Vogel et al, 1993; Pyrhönen et al, 1994). At a dose of 40–240 mg day$^{-1}$, TOR has been well tolerated as first-line treatment in advanced ER-positive or undetermined breast cancer. The response rates have ranged between 43% and 61% (Valavaara et al, 1988; Hietanen et al, 1990; Tominaga et al, 1993). Consequently, a few large phase III studies were initiated comparing tamoxifen with toremifene (Pyrhönen, 1990; Hayes et al, 1995).

The aim of the present study was to compare the safety and efficacy of TOR 60 mg day$^{-1}$ with TAM 40 mg day$^{-1}$ in the treatment of post-menopausal patients with advanced breast cancer.

### PATIENTS AND METHODS

### Study design

This study is a double-blind, parallel group, randomized multicentre clinical study. A total of 415 women were enrolled in 26 centres in six countries. The study was initiated in Finland in 1986. Later, it was extended to Sweden, Norway, Poland, Hungary and the Czech Republic.

### Patient eligibility

Eligible patients were post-menopausal (1 year since the last menstruation or age 55 years if a hysterectomy had been performed) and had histologically or cytologically verified inoperable primary, metastatic or recurrent breast cancer with at least one measurable or evaluable (bone disease) lesion. ER status had to be positive or unknown. Prior adjuvant therapy was allowed if at least 12 months had elapsed since the last endocrine treatment or the patient had fully recovered from cytotoxic therapy. The anticipated survival had to be at least 3 months and the Karnofsky performance status ≥ 50%. Patients were excluded if they had severe concomitant renal, hepatic, cardiac, diabetic or thromboembolic diseases or other malignancies; basal cell cancer of the skin or cervical carcinoma in situ were allowed. Continuous use of corticosteroids for diseases other than cancer was allowed only if it had been started 6 months before randomization. Patients who were not considered able to cooperate were also excluded. Written or documented verbal informed consent was required. The study was approved by the ethics committees in each participating hospital.

### Randomization and treatment

The patients were stratified by whether or not they had measurable or only evaluable disease and were randomly assigned to treatment with TOR or TAM. Randomization was performed centrally, using computer generated lists that were prepared separately for both stratification groups and for each participating centre.

The study medications were provided by Orion-Farmos as tablets containing either 60 mg of TOR or 40 mg of TAM. The dose of TAM was chosen on the basis of clinical practice in Scandinavia and the dose of TOR was based on the previous phase II results (Valavaara et al, 1988). The dosage for both arms was one tablet by mouth daily, preferably at breakfast time. The treatment continued until disease progression or adverse events precluded the use of the drug. Minimum treatment period was 2 months. The patients were required to return all unused tablets, which were counted and recorded to assess treatment compliance.

### Patient evaluation and response criteria

All patients had a complete history and physical examination no earlier than 4 weeks before the treatment: blood chemistry, haemoglobin, a leucocyte and platelet count, a chest radiograph, a skeletal scintigraphy, an abdominal sonography or computerized tomography (CT) scan and assessment of performance status. If the scintigraphy revealed bone metastasis, a radiograph was then obtained to verify the finding. All lesions, signs and symptoms were registered.

The follow-up examinations were performed bimonthly for 2 years and thereafter at 4-month intervals. At each follow-up a careful clinical examination was done and the same blood tests were taken as at baseline. In case of multiple bone metastasis, at least four and invariably more than 30% of the lesions were evaluated bimonthly except for the skeletal scintigraphy which was taken every fourth month. In case of a negative baseline result, a chest radiograph, an abdominal sonography or CT scan and skeletal scintigraphy were performed twice annually. All adverse events were registered and the degree of causality to the study drug was evaluated.

The study met the GCP standards with 100% source data verification during the study. The responses were evaluated according to the WHO criteria adopted by the UICC (Miller et al, 1981). Treatment response (CR, PR, NC) was accepted only if confirmed at two consecutive evaluations 2 months apart. All objective responses and the duration of the response were re-evaluated and approved by the investigator's review board. Time to progression (TTP) was defined as the time between randomization and onset of relapse or disease progression. Patients who had discontinued the study prematurely and patients who were still continuing in the study were treated as censored observations from the time of randomization until the time of discontinuation, or until the time of the latest follow-up visit (before 31 December 1993) respectively. Time to treatment failure (TTF) was defined as the period from the day of randomization to the day when disease progression, treatment-related toxicity, resulting in discontinuation of therapy or death (from any cause) occurred.

© Cancer Research Campaign 1997 

**Table 1** Patient characteristics

| | Treatment arm | |
|---|---|---|
| | Toremifene ($n$ = 214) | Tamoxifen ($n$ = 201) |
| Characteristic | | |
| Mean age (range)[a] | 65.5 (33.6–87.6) | 65.9(44.8–90.2) |
| Mean disease free interval (range)[a] | 4.7 (0.0–32.9) | 4.0 (0.0–33.8) |
| Mean number of organ sites (range)[b] | 2.2 (1.0–7.0) | 2.3 (1.0–8.0) |
| Stratificational group[b] | | |
| Measurable | 163 (76.2)[c] | 152 (75.6) |
| Non-measurable | 49 (22.9) | 45 (22.4) |
| Dominant site of disease[b] | | |
| Visceral | 60 (28.0) | 63 (31.3) |
| Bone | 68 (31.8) | 70 (34.8) |
| Soft tissue | 84 (39.3) | 64 (31.8) |
| Karnofsky Performance status[d] | | |
| 100 | 58 (27.1) | 50 (24.9) |
| 80–90 | 117 (54.7) | 111 (55.2) |
| 60–70 | 32 (15.0) | 33 (16.4) |
| 40–50 | 6 (2.8) | 6 (3.0) |
| Prior hormonal therapy[e] | | |
| No | 201 (93.9) | 183 (91.0) |
| Yes | 13 (6.1) | 17 (8.5) |
| ER status | | |
| Known | 121 (56.5) | 117 (58.2) |
| Unknown | 93 (43.5) | 84 (41.8) |
| PR status | | |
| Known | 109 (50.9) | 112 (55.7) |
| Unknown | 105 (49.1) | 89 (44.3) |

[a]In years, data not available for zero and one patient respectively; [b]data not available for two and four patients respectively; [c]number (%) of patients; [d]data not available for one and one patients respectively; [e]Data not available for zero and one patient respectively.

**Table 2** Response rates to toremifene and tamoxifen

| | Treatment arm | |
|---|---|---|
| | Toremifene | Tamoxifen |
| *All randomized patients* | **($n$ = 214)** | **($n$ = 201)** |
| Complete response | 19 (8.9)[a] | 19 ( 9.5) |
| Partial response | 48 (22.4) | 56 (27.9) |
| Stable disease | 50 (23.4) | 52 (25.9) |
| Progressive disease | 83 (38.8) | 55 (27.4) |
| Not evaluable | 14 ( 6.5) | 19 ( 9.5) |
| Complete and partial response | 67 (31.3) | 75 (37.3) |
| *Eligible and evaluable patients* | **($n$ = 186)** | **($n$ = 173)** |
| Complete response | 18 ( 9.7) | 18 (10.4) |
| Partial response | 46 (24.7) | 53 (30.6) |
| Stable disease | 47 (25.3) | 51 (29.5) |
| Progressive disease | 75 (40.3) | 51 (29.5) |
| Complete and partial response | 64 (34.4) | 71 (41.0) |

[a]Number (%) of patients.

### Statistics

A total of 344 evaluable patients were required in order to detect a 15% difference in response rates between TOR and TAM with a two-sided type I error rate ($\alpha$) of 0.05 and a type II error rate ($\beta$) of 0.20. The assessment of treatment efficacy was based on the two primary variables, that is response rate and time to progression. A conservative testing procedure was obtained by demanding a statistically significant testing result at the 0.05 level for both of these variables or at the 0.025 level for one of them, in case the other one remained statistically insignificant.

All data were independently verified for correctness of entry and subjected to both manual and computerized checks for logic and consistency before being made available for statistical analysis. Efficacy and safety were assessed by the intent to treat principle when at least 70% of the enrolled patients had experienced progressive disease. The corresponding data cut-off date was 31 December 1993.

The comparisons between TOR and TAM arms with respect to qualitative variables were made using either Fisher's exact test or Pearson's chi-square test. In case of quantitative variables Wilcoxon's rank-sum test was applied. The log-rank test was used to compare the two treatment arms with regard to duration variables (i.e. TTP, TTF, response duration and overall survival) and Cox's regression model was fitted for each of these variables to obtain an estimate for the hazard ratio (TAM/TOR) and the corresponding 95% confidence interval (CI).

### RESULTS

### Patient characteristics

Between June 1986 and May 1992, 415 patients (TOR 214, TAM 201) were accrued. Nineteen patients in the TOR arm (ER-negative tumour, four patients; non-measurable disease other than bone, three patients each; premenopausal patients, two patients; inadequate baseline examinations two; other eight) and 17 in the TAM arm (non-measurable disease other than bone, five patients; ER-negative tumour, three patients; the concurrent presence of a second active malignancy, three patients; other six) were ineligible. The median follow-up period of the patients was 25.2 months. The characteristics of the patient populations before treatment are listed in Table 1. These characteristics are evenly balanced between the two arms except for the levels of ER receptors: in the TAM group a larger proportion of patients had high ER levels, which is reflected by mean ER concentrations (119 and 171 fmol $mg^{-1}$ cytosol protein for TOR and TAM patients respectively).

### Response

Response data are presented in Table 2. The response rates were calculated for all randomized patients as well as for all eligible and all evaluable patients separately. There were 19 complete responses in both treatment arms and 48 vs 56 partial responses with TOR and TAM arm respectively. The rates of complete plus partial responses for TOR and TAM were 31.3% and 37.3% respectively. The difference was not statistically significant ($P$ = 0.215). The 95% CI for the difference was –15.1% to 3.1%.

The median time to the onset of objective response for all randomized patients was 3.9 months with TOR and 2.6 months with TAM ($P$ = 0.455). The median duration of complete and

© Cancer Research Campaign 1997

**Table 3** Time to progression, time to treatment failure and overall survival of patients on toremifene and tomoxifen (all randomized patients)

| | Treatment arm | |
|---|---|---|
| | **Toremifene (n = 214)** | **Tamoxifen (n = 201)** |
| Median (mean) time to progression[a,b] | 7.3 (13.0) | 10.2 (15.3) |
| Number (%) of patients with PD | 176 (82.2) | 147 (73.1) |
| Median (mean) time to treatment failure[a] | 6.3 (12.2) | 8.5 (12.7) |
| Number (%) of patients failed | 195 (91.1) | 181 (90.0) |
| Median (mean) overall survival[a] | 33.0 (37.5) | 38.7 (37.5) |
| Number (%) of patients dead | 123 (57.5) | 115 (57.2) |

[a]In months from randomization; [b]$P = 0.047$

**Figure 1** Time to progression among toremifene (——, *n* = 214) and tamoxifen (. . ., *n* = 201) treated patients (*P* = 0.047, log-rank test)

**Figure 2** Time to progression among toremifene (——, *n* = 121) and tamoxifen (. . ., *n* = 117) treated oestrogen receptor-positive patients. (*P* = 0.578, log-rank test)

**Figure 3** Overall survival among toremifene (——, *n* = 214) and tamoxifen (. . ., *n* = 201) treated patients (*P* = 0.645, log-rank test). Survival time was defined as the time between randomization and death. Patients who were alive were treated as censored observations from the time of randomization until the last date they were known to be alive (before 31 December 1993)

partial responses were 26.3 and 18.3 months for TOR and 26.4 and 18.4 months for TAM respectively. There was no significant difference in either response group (CR: $P$ = 0.224; PR: $P$ = 0.706). Lesions in different sites responded similarly to both treatments (data not shown).

### Time to progression and time to treatment failure

At the time of data cut-off, 19 (8.9%) patients were continuing on the study without evidence of disease progression on TOR and 20 (10%) on TAM. The results of TTP and TTF are presented in Table 3 and Figures 1 and 2. There was a transient difference between the TTP curves, which approached statistical significance, favouring TAM over TOR ($P$ = 0.047). The median TTP was 7.3 months (95% CI 5.8–8.8) for TOR patients and 10.2 months (95% CI 8.1–12.5) for TAM. The hazard ratio for disease progression was 0.801 (95% CI 0.640–1.00). The mean TTPs were 13.0 and 15.3 months for TOR and TAM respectively. Owing to the higher incidence of treatment discontinuations in the TAM arm, there was no difference in TTF between TOR and TAM (median 6.3 and 8.5 and mean 12.2 and 12.7 respectively; $P$ = 0.271). Hazard ratio for TTF was 0.89 (95% CI 0.73–1.09).

Further analysis confirmed that the slight difference of TTP may be due to an imbalance in the prognostic factors, as the outcome of the two treatments was similar in patients with ER-positive tumours (Figure 2) and the difference was mainly due to the effects encountered in patients with tumours of unknown ER status. For these patients the median TTP on TOR was 6.3 months (95% Cl 4.4–8.0) and on TAM 11.4 months (95% Cl 8.1–14.4) ($P$ = 0.019), whereas for the patients with ER-positive tumours the TTP was 9.1 (95% CI 4.6–11.6) and 10.1 months (95% Cl 6.5–12.5) ($P$ = 0.578). With regard to TTF, the ER status yielded analogous $P$-values of 0.103 and 0.984 respectively.

### Survival

Of the 415 randomized patients, 238 had died by the data cut-off date; 123 patients in the TOR arm and 115 in the TAM arm. Survival data are presented in Table 3 and Figure 3. The median survival time for patients treated with TOR was 33.0 months (95% CI 28.5–37.0) and 38.7 months (95% CI 31.8–41.9) for those treated with TAM ($P$ = 0.645).

© Cancer Research Campaign 1997

**Table 4** Adverse events associated with toremifene and tamoxifen (treatment related or indeterminate cause)

| Event | Treatment arm | |
|---|---|---|
| | Toremifene ($n$ = 214) | Tamoxifen ($n$ = 201) |
| Sweating | | |
| All | 31 (14.5)[a] | 23 (11.4) |
| Moderate/severe[b] | 8 (3.7) | 3 (1.5) |
| Hot flaushes | | |
| All | 24 (11.2) | 21 (10.4) |
| Moderate/severe | 5 (2.3) | 8 (4.0) |
| Nausea | | |
| All | 12 (5.6) | 19 (9.5) |
| Moderate/severe | 3 (1.4) | 6 (3.0) |
| Vaginal discharge | | |
| All | 12 (5.6) | 7 (3.5) |
| Moderate/severe | 3 (1.4) | 0 (0.0) |
| Oedema | | |
| All | 4 (1.9) | 6 (3.0) |
| Moderate/severe | 2 (0.9) | 1 (0.5) |
| Dizziness | | |
| All[c] | 3 (1.4) | 10 (5.0) |
| Moderate/severe[d] | 0 (0.0) | 5 (2.5) |
| Vomiting | | |
| All | 3 (1.4) | 3 (1.5) |
| Moderate/severe | 3 (1.4) | 2 (0.9) |
| Vaginal bleeding | | |
| All | 2 (0.9) | 3 (1.5) |
| Moderate/severe | 0 (0.0) | 1 (0.5) |

[a]Number (%) of patients; [b]by WHO severity grading; [c]$P$ = 0.048; [d]$P$ = 0.026.

**Table 5** Premature study discontinuations

| Reason | Treatment arm | |
|---|---|---|
| | Toremifene ($n$ = 214) | Tamoxifen ($n$ = 201) |
| Death | 4 (1.9)[a] | 10 (5.0) |
| Lost to follow-up | 1 (0.5) | 6 (3.0) |
| Other therapy | 5 (2.3) | 7 (3.5) |
| Intercurrent illness | 5 (2.3) | 4 (2.0) |
| Adverse event/refusal | 2 (0.9) | 7 (3.5) |
| Protocol violation | 2 (0.9) | 0 (0.0) |
| Other | 2 (0.9) | 4 (2.0) |
| Total[b] | 21 (9.8) | 38 (18.9) |

[a]Number (%) of patients; [b]$P$ = 0.011.

### Toxicity

Overall, TAM therapy was associated with a slightly higher frequency of adverse events than TOR (44.3% vs 39.3%). The major adverse events are presented in Table 4. Most of the adverse events were mild or moderate. Only four patients (three sweating, one hot flushes, nausea, vomiting and oedema) on TOR and six patients (four nausea, one sweating, one vaginal bleeding) on TAM experienced severe adverse events. Moderate dizziness was reported significantly more often ($P$ = 0.026) among TAM patients than among TOR patients.

**Table 6** Other treatment emergent adverse events

| Event | Treatment arm | |
|---|---|---|
| | Toremifene ($n$ = 214) | Tamoxifen ($n$ = 201) |
| Thromboembolic events | 11 (5.1)[a] | 11 (5.5) |
| Cardiac events | 7 (3.3) | 7 (3.5) |
| Ocular abnormalities[b] | 3 (1.4) | 7 (3.5) |
| Neoplasms | 5 (2.3) | 8 (4.0) |
| Elevated liver function tests[c] | | |
| Alkaline phosphatase | 13 (6.0) | 16 (8.0) |
| SGOT[d] | 6 (2.8) | 15 (7.5) |
| Total bilirubin | 0 (0.0) | 2 (1.0) |
| Elevated calcium[e] | 0 (0.0) | 0 (0.0) |

[a]Number (%) of patients; [b]0 (0.0) and 5 (2.5) cataracts respectively, $P$ = 0.026; [c]abnormality criteria: > 2.5 times the upper limit of reference range; [d]$P$ = 0.042; [e]abnormality criteria: > 1.25 times the upper limit of reference range.

**Table 7** Deaths during study

| Cause of death[a] | Treatment arm | |
|---|---|---|
| | Toremifene ($n$ = 214) | Tamoxifen ($n$ = 201) |
| Disease progression | 10 (4.7)[b] | 9 (4.5) |
| Other malignancies | 0 (0.0) | 1 (0.5) |
| Respiratory system disorders | 1 (0.5) | 4 (2.0) |
| Vascular disorders | 3 (1.4) | 0 (0.0) |
| Gastrointestinal system disorders | 0 (0.0) | 1 (0.5) |
| Platelet, bleeding disorders | 0 (0.0) | 1 (0.5) |
| Other | 0 (0.0) | 4 (2.0) |
| Total | 14 (6.5) | 20 (10.0) |

[a]By WHO system organ class; [b]number (%) of patients.

The reasons for premature study drug discontinuations are presented in Table 5. A total of 21 (9.8%) patients discontinued TOR and 38 patients (18.9%) TAM, for various reasons ($P$ = 0.011). Two of the TOR patients (0.9%) vs seven of the TAM patients (3.5%) discontinued the treatment due either to toxicity or to refusal.

Other treatment emergent adverse events are listed in Table 6. Five patients developed cataracts while on TAM and none on TOR ($P$ = 0.026). Of the 13 other malignancies detected before the data cut-off date, five were found among TOR patients and eight among TAM patients. One endometrial carcinoma and two ovarian cyst malignancies were observed among TAM patients, but no gynaecological malignancies among TOR patients. Otherwise, the distribution of other malignancies was very similar. Elevated liver enzyme, SGOT, was observed more frequently among TAM patients than TOR patients ($P$ = 0.042). The difference was still of borderline significance ($P$ = 0.082), when excluding the one patient in TOR and three patients in the TAM group with liver metastases.

There was no difference concerning fatal disorders (Table 7) during or within 1 month after discontinuation of the treatment. The majority of the deaths were due to metastatic breast cancer and not related to treatment itself. There were no deaths directly associated with the study drugs.

© Cancer Research Campaign 1997

The performance status and body weights of the patients were similar in the treatment groups throughout the study.

### DISCUSSION

This multicentre phase III study with two parallel groups compared the efficacy and safety profile of TOR (60 mg daily) with TAM (40 mg daily) as first-line treatment of inoperable primary or metastatic ER-positive or undetermined breast cancer in post-menopausal women. This is the only large-scale double-blind trial comparing these two drugs (Pyrhönen, 1990). The primary study variables were response rate, TTP, overall survival and safety. In addition, variables such as TTF, time to onset of response and response duration were followed. The response rate was slightly higher for patients on TAM, but the difference was not statistically significant. The strict criteria for efficacy assessment and the great proportion of patients (43%) with tumours of unknown ER status may explain the relatively low proportion of objective responses in both treatment groups compared with those encountered in the earlier phase II studies with TOR (Valavaara et al, 1988; Hietanen et al, 1990; Tominaga et al, 1993).

At the time of closing the database for analysis, 19 patients continued the treatment with TOR and 20 with TAM. The TTP plot was in favour of TAM, but suggests a transient, rather than a persistent difference between the treatment groups. Subgroup analysis shows that the difference was essentially generated in the patient group with tumours of unknown ER status. There was no difference between the treatment groups if the patient population with ER-positive tumours ($n = 238$) is considered separately. There was, evidently, some major imbalance in receptor levels or some other prognostic factors among the patients with unknown ER status when compared with ER-positive tumour patients, because the TTP was longer in the ER unknown group of TAM patients (median 11.4 vs 10.1 months). As expected, the TTP was shorter in TOR-treated patients with ER-unknown than ER-positive tumours (median 6.3 vs 9.1 months). In previous studies, positive correlation between ER concentration and TTP as well as response duration has been reported for various hormonal treatments of breast cancer (Bonomi et al, 1988; Valavaara et al, 1990). In addition, the ER concentrations were higher in the TAM patients with known ER status in the present study. Considering that more patients on TAM than on TOR discontinued the study prematurely, the TTF did not differ between the treatment groups.

At the time of closing the database, 18 patients with objective response continued the study on TOR and 16 on TAM. There were no significant differences between the treatment groups as regards response duration or survival.

The present study indicates that in the treatment of metastatic breast cancer TAM and TOR are equally safe and effective, confirming the results of another large randomized trial (Hayes et al, 1995). The toxicity was mostly mild or moderate and usually attributable to the antioestrogenic/oestrogenic properties of the study drugs. Significantly more patients experienced treatment-emerged dizziness and cataract in the TAM group than the TOR group. As no regularly performed eye examinations were organized in the study, the difference in the frequency of cataracts must be taken into account with great reservation. Considering previously reported ocular toxicity of tamoxifen (Gerner, 1989; Pavlidis et al, 1992; Heier et al, 1994), this should be further explored prospectively in other trials. The higher incidence of treatment discontinuations due to toxicity or patient refusal in the TAM group, may also reflect the somewhat higher toxicity of TAM in the dose used in this study in comparison with TOR. Overall, however, both treatments were well tolerated.

Preclinical toxicity data show that TOR is not carcinogenic, whereas TAM has strong hepatocarcinogenicity in rats, which suggests, potentially, an important safety advantage for TOR. In a 2-year mouse carcinogenicity study, TOR induced tumours in the ovaries, bone and testes (to be published elsewhere). The corresponding 2-year study with TAM is not available. Because oestrogens cause similar tumours in mice, and TOR is mainly oestrogenic in this species, these tumours are considered to be species specific and to be of little clinical relevance to humans.

The widespread use of TAM in adjuvant medical therapy and even as a chemopreventive agent has raised the question of its safety in view of its carcinogenicity in animals. In adjuvant trials, TAM has been shown to increase the incidence of uterine cancers (Fornander et al, 1993; Fisher et al, 1994; De Gregorio et al, 1995; Rutqvist et al, 1995), although the median follow-up times have been less than 10 years. The expected time from tumour initiation to clinically detectable cancer has been considered to be between 10 and 15 years. The mechanism of TAM-induced carcinogenicity may be partly hormonal (Metzler and Degen, 1987; Williams et al, 1993), i.e. promotion of existing tumours, and partly non-hormonal, i.e. initiation of tumours or mutagenesis of p53 tumour suppressor gene (Styles et al, 1994; Vancutsem et al, 1994). As TAM induces DNA adducts and is carcinogenic in the rat liver (Han and Liehr, 1992; Hard et al, 1993; Hirsimäki et al, 1993; Montandon et al, 1994), non-hormonal mechanisms might be preferentially related to the initiation of secondary malignancies. The recent report that TAM-treated patients have an increased incidence of gastrointestinal tumours is disconcerting (Rutqvist et al, 1995). Studies have now been initiated to compare the genotoxic potential of TOR with TAM in adjuvant therapy of breast cancer.

In conclusion, the results of this double blind trial suggest that TOR (60 mg day$^{-1}$) and TAM (40 mg day$^{-1}$) are equally effective in the treatment of advanced ER-positive or ER-unknown breast cancer in post-menopausal patients. The safety profile of TOR makes it an attractive alternative to TAM, especially in long-term treatment of breast cancer. The on-going comparative trials on these drugs as an adjuvant treatment will further elucidate the feasibility of TOR in lesser stages of breast cancer. Exploration on chemoprevention with TOR should also be considered.

### ACKNOWLEDGEMENTS

We are greatly indebted to professors Sándor Eckkard, Lars R Holsti, Stener Kvinnsland, Eeva Nordman, Pentti Rissanen, Pentti Taskinen, and the late professor Josef Zborzil, who never saw this study completed. We also want to acknowledge Ms Päivi Nevala, the clinical monitor, and Ms Raija Vassinen, the study nurse, for their excellent assistance and important contribution to the study.

This trial was supported by Orion Corporation Orion-Farmos Pharmaceuticals.

### REFERENCES

Bonomi P, Gale M, von Roenn J, Andersson K, Johnson P, Wolter J and Wolter J (1988) Quantitative estrogen and progesterone receptor levels related to progression free interval in advanced breast cancer patients treated with megesterol acetate or tamoxifen. *Sem Oncol* **15** (suppl. 1): 26–33

© Cancer Research Campaign 1997

De Gregorio MW, Mäenpää JV and Wiebe VJ (1995) Tamoxifen for the prevention of breast cancer. In *Important Advances in Oncology*, De Vita VT, Hellman S and Rosenberg SA (eds), pp.175–185. Lippincott: Philadelphia

Early Breast Cancer Trialists' Collaborative Group (1992) Systemic treatment of early breast cancer by hormonal, cytotoxic, or immune therapy. 133 randomised trials involving 31,000 recurrences and 24,000 deaths among 75,000 women. *Lancet* **339**: 71–85

Fisher B, Constantino JP, Redmond CK, Fisher ER, Wickerham DL and Cronin WM (1994) Endometrial cancer in tamoxifen treated breast cancer patients: findings from the National Surgical Adjuvant Breast and Bowel Project (NSABP) B-14. *J Natl Cancer Inst* **86**: 527–537

Fornander T, Hellström A-C and Moberger B (1993) Descriptive clinicopathologic study of 17 patients with endometrial cancer during or after adjuvant tamoxifen in early breast cancer. *J Natl Cancer Inst* **85**: 1850–1855

Gerner EW (1989) Ocular toxicity of tamoxifen. *Ann Ophthalmol* **21**: 420–423

Hamm JT, Tormey DC, Kohler PC, Haller D, Green M and Shemano I (1991) Phase I study of toremifene in patients with advanced cancer. *J Clin Oncol* **9**: 2036–2041

Han Y and Liehr JG (1992) Induction of covalent DNA adducts in rodents by tamoxifen. *Cancer Res* **52**: 1360–1363

Hard GC, Iatropoulos MJ, Jordan K, Radi L, Kaltenberg OP, Imondi A and Williams G (1993) Major difference in the hepatocarcinogenicity and DNA adduct forming ability between toremifene and tamoxifen in femal Crl: CD (BR) rats. *Cancer Res* **53**: 4534–4541

Hayes D, Van Zyl JA, Hacking A, Goedhals L, Bezwoda WR, Mailliard JA, Jones SE, Vogel CL, Berris RF, Shemano I and Schoenfelder J (1995) Randomized comparison of tamoxifen and two separate doses of toremifene in postmenopausal patients with metastatic breast cancer. *J Clin Oncol* **13**: 2556–2566

Heier JS, Dragoo RA, Enzenauer RW and Waterhouse WJ (1994) Screening for ocular toxicity in asymptomatic patients treated with tamoxifen. *Am J Ophthalmol* **117**: 772–775

Hietanen T, Baltina D, Johansson R, Numminen S, Hakala T, Helle L and Valavaara R (1990) High dose toremifene (240 mg daily) is effective as first line hormonal treatment in advanced breast cancer – an ongoing phase II multicenter Finnish–Latvian cooperative study. *Breast Cancer Res Treat* **16** (suppl.): S37–S40

Hirsimäki P, Hirsimäki Y, Nieminen L and Payne BJ (1993) Tamoxifen induces hepatocellular carcinoma in rat liver: a 1-year study with two antiestrogens. *Arch Toxicol* **67**: 49–54

Jaiyesimi IA, Buzdar AU, Decker DA and Hortobagyi GN (1995) Use of tamoxifen for breast cancer: Twenty-eight years later. *J Clin Oncol* **13**: 513–529

Jönsson P-E, Malmberg M, Bergljung L, Ingvar C, Ericsson M, Ryden S, Nilsson I and Johansson Terje I (1991) Phase II study of high dose toremifene in advanced breast cancer progressing during tamoxifene treatment. *Anticancer Res* **11**: 873–876

Kallio S, Kangas L, Blanco G, Johansson R, Karjalainen A, Perilä M, Piippo I, Sundqvist H, Södervall M and Toivola R (1986) A new triphenylethylene compound, FC-1157a. II Hormonal effects. *Cancer Chemother Pharmacol* **17**: 103–108

Karlsson S, Hirsimäki Y, Mäntylä E, Nieminen L, Kangas L, Hirsimäki P, Perry CJ, Mulhern M, Millar P, Handa J and Williams GM (1996) A two-year dietary carcinogenicity study of the antiestrogen toremifene in Sprague–Dawley rats. *Drug Chem Toxi* **19**: 245–266

Kangas L (1992*a*) Agonistic and antagonistic effects of antiestrogens in different target organs. *Acta Oncol* **31**: 143–146

Kangas L (1992*b*) General pharmacology and applicability of toremifene, a new antiestrogenic antitumour drug. *Eur J Surg Oncol* **18** (suppl. 1): 23

Kangas L, Nieminen A-L, Blanco G, Grönroos M, Kallio S, Karjalainen A, Peril M, Södervall M and Toivola R (1986) A new triphenylethylene compound, FC-1157a. II. Antitumour effects. *Cancer Chemother Pharmacol* **17**: 109–113

Kivinen S and Mäenpää J (1990) Effect of toremifene on clinical chemistry, hematology and hormone levels at different doses in healthy postmenopausal volunteers: phase I study. I. *Steroid Biochem* **36**: 217–220

Lahti E, Blanco G, Kauppila A, Apaja-Sarkkinen M, Taskinen P and Laatikainen T (1993) Endometrial changes in postmenopausal breast cancer patients receiving tamoxifen. *Obstet Gynecol* **81**: 660–664

Metzler M and Degen GH (1987) Sex hormones and neoplasia: liver tumours in rodents. *Arch Toxicol* **10** (suppl.): 251–263

Miller AB, Hoogstraten B, Staquet M and Winkler A (1981) Reporting results of cancer treatment. *Cancer* **47**: 207–214

Montandon F and Williams GM (1994) Comparison of DNA reactivity of the polyphenylethylene hormonal agents diethylstilbestrol, tamoxifen and toremifene in rat and hamster liver. *Arch Toxicol* **68**: 272–275

Pavlidis NA, Petris C, Briassoulis E, Klouvas G, Psilas C, Rempapis J and Petroutsos G (1992) Clear evidence that long-term, low-dose tamoxifen treatment can induce ocular toxicity. *Cancer* **69**: 2961–2964

Pyrhönen S (1990) Phase III studies of toremifene in metastatic breast cancer. *Breast Cancer Res Treat* **16** (suppl): S41–S46

Pyrhönen S, Valavaara R, Heikkinen M, Rissanen P, Blanco G, Nordman E, Holsti LR and Hajba A (1990) Treatment of advanced breast cancer with 20 mg toremifene, a phase II study. Preliminary communication. *J Steroid Biochem* **36**: 227–228

Pyrhönen S, Valavaara R, Vuorinen J and Hajba A (1994) High dose toremifene in advanced breast cancer resistant to or relapsed during tamoxifen treatment. *Breast Cancer Res Treat* **29**: 223–228

Rutqvist L-E, Johansson H, Signomklao T, Johansson U, Fornander T and Wilking N (1995) Adjuvant tamoxifen therapy for early stage breast cancer and second primary malignancies. *J Natl Cancer Inst* **87**: 645–651

Santen RJ, Manni A, Harvey H and Redmond C (1990) Endocrine treatment of breast cancer in women. *Endocrine Rev* **11**: 221–265

Styles JA, Davies A, Lim CK, De Matteis F, Stanley LA, White INH, Yan Z-X and Smith LL (1994) Genotoxicity of tamoxifen, tamoxifen epoxide and toremifene in human lymphoblastoid cells containing human cytochrome P450s. *Carcinogenesis* **15**: 5–9

Tominaga T, Abe O, Izuo M and Nomura Y (1993) Phase II study of NK 622 (toremifene citrate) in advanced breast cancer, a multicentral cooperative dose finding study. *Gan To Kagaku Ryoho* **20**: 79–90

Valavaara R and Pyrhönen S (1989) Low-dose toremifene in the treatment of estrogen-receptor-positive advanced breast cancer in postmenopausal women. *Curr Therap Res* **46**: 966–973

Valavaara R, Pyrhönen S, Heikkinen M, Rissanen P, Blanco G, Thölix E and Nordman E (1988) Toremifene, a new antiestrogenic compound for treatment of advanced breast cancer. Phase II study. *Eur J Cancer Clin Oncol* **24**: 785–790

Valavaara R, Tuominen J and Johansson R (1990) Predictive value of tumour estrogen and progesterone receptor levels in postmenopausal women with advanced breast cancer treated with toremifene. *Cancer* **66**: 2264–2269

Vancutsem PM, Lazarus P and Williams GM (1994) Frequent and specific mutations of the rat P53 gene in hepatocarcinomas induced by tamoxifen. *Cancer Res* **54**: 3864–3867

Vogel CL, Shemano I, Schoenfelder J, Gams RA and Green MR (1993) Multicenter phase II efficacy trial of toremifene in tamoxifen-refactory patients with advanced breast cancer. *J Clin Oncol* **11**: 345–350

Vuorio T, Wärri A, Sandberg M, Alitalo K and Vuorio E (1988) Expression of the c-Ha-ras and neu oncogenes in DMBA-induced, antiestrogen-treated rat mammary tumours. *Int J Cancer* **42**: 774–779

Wärri AM, Huovinen RL, Laine AM, Martikainen PM and Härkönen PL (1993) Apoptosis in toremifene-induced growth inhibition of human breast cancer cells in vivo and in vitro. *J Natl Cancer Inst* **85**: 1412–1418

White IN, De Matteis F, Davies A, Smith LL, Crofton-Sleigh C, Venitt S, Hewer A and Phillips DH (1992) Genotoxic potential of tamoxifen and analogues in female Fischer F344 /n rats, DBA/2 and C57BL/6 mice and in human MCL-5 cells. *Carcinogenesis* **13**: 2197–2203

Williams GM, Iatropoulos M, Cheung R, Radi L and Wang CX (1993) Diethylstilbestrol liver carcinogenicity and modification of DNA in rats (letter). *Cancer* **68**: 193–198

Zito R (1994) Problems in breast cancer prevention and therapy with tamoxifen. *J Exp Clin Cancer Res* **13**: 99–102

### APPENDIX

The complete list of investigators and participating institutions (number of patients)

Finland: S Pyrhönen, University Central Hospital Helsinki (92); R Valavaara, University Central Hospital Turku (52); G Blanco, S Virtanen, M Heikkinen, University Hospital Oulu (14); T Hietanen, University Hospital Tampere (12); R Johansson, University Hospital Kuopio (7); Sweden: H Modig, L Hardell, Regional Hospital Umeå (23); G Westman, J Ahlgren, Regional Hospital Örebro (18); I Nilsson, Central Hospital Malmö (13); H Sellström, Säffle Hospital (5); U Ljungqvist, Regional Hospital Falun (4); U Glas, E Lidbrink, Södersjukhuset of Stockholm (4); J Järhult, Regional Hospital Eksjö (3); P-E Jösson, Regional Hospital Helsingborg (1); Norway: S Gundersen, L Ottestad,

© Cancer Research Campaign 1997

Norwegian Radiumhospital Oslo (29); S Lundgren, S Kvinnsland, Central Hospital Trondheim (15); O Mella, Haukeland Hospital Bergen (13); Poland: M Pawlicki, J Rolski, Marie Sklodowska-Curie Memorial Institute Cracow (35); J Zborzil, T Pienkowski, Oncological Institute Warsaw (22); Czech Republic: J Bauer, I Krajsová, O Pribylová, Charles University Hospital Prague (20); I Bustová, Ceské Budejovice Hospital (9); M Svehelka, Therapeutical Institute, Ples Hospital (4); Hungary: I Hindy, National Institute of Oncology Budapest (8); G László, Veszprem County Hospital (8); K Moskovits, Tétényi Hospital Budapest (6); M Osváth, Komárom County Hospital Tatabánya (6); L Perényi, Szeged University Hospital (1).

*© Cancer Research Campaign 1997*